# Supplementary material for: Association between oral microbiome and seven types of cancers in East Asian population: a two-sample Mendelian randomization analysis
Source: Front Mol Biosci. 2023 Nov 21;10:1327893. doi: 10.3389/fmolb.2023.1327893 (PMC10702768; doi:10.3389/fmolb.2023.1327893)
Supplement: Supplementary file 1 [file DataSheet1.ZIP › Supplementary Materials/MR plots of saliva/saliva═╝/Lung Cancer/pheno.2012_to_lung cancer_forest.pdf]

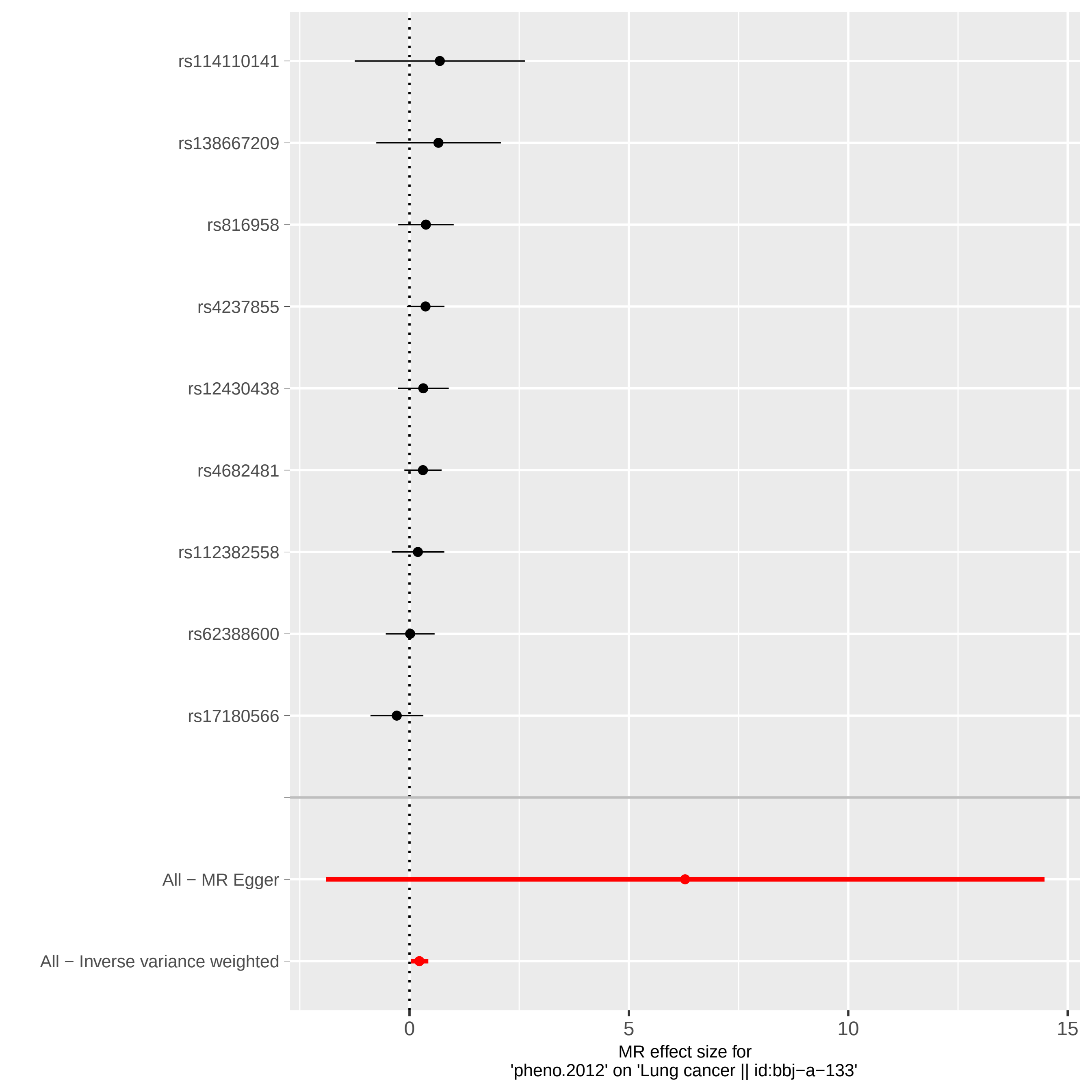
rs114110141
rs138667209
rs816958
rs4237855
rs12430438
rs4682481
rs112382558
rs62388600
rs17180566
All − MR Egger
All − Inverse variance weighted
0
5
10
15
MR effect size for
'pheno.2012' on 'Lung cancer || id:bbj−a−133'
